# The Effect of Functional Gradient Material Distribution and Patterning on Torsional Properties of Lattice Structures Manufactured Using MultiJet Fusion Technology

**DOI:** 10.3390/ma14216521

**Published:** 2021-10-29

**Authors:** Yeabsra Mekdim Hailu, Aamer Nazir, Shang-Chih Lin, Jeng-Ywan Jeng

**Affiliations:** 1 High Speed 3D Printing Research Center, National Taiwan University of Science and Technology, No. 43, Section 4, Keelung Road, Taipei 10607, Taiwan; M10803811@mail.ntust.edu.tw (Y.M.H.); xyz@mail.ntust.edu.tw (S.-C.L.); 2 Department of Mechanical Engineering, National Taiwan University of Science and Technology, No. 43, Section 4, Keelung Road, Taipei 10607, Taiwan; 3 Department of Industrial and Systems Engineering, The Hong Kong Polytechnic University, Hung Hom, Kowloon, Hong Kong SAR 999077, China; 4 Graduate Institute of Biomedical Engineering, National Taiwan University of Science and Technology, No. 43, Section 4, Keelung Road, Taipei 10607, Taiwan; 5 President Office, Lunghwa University of Science and Technology, No.300, Section 1, Wanshou Rd. Guishan District, Taoyuan City 333326, Taiwan

**Keywords:** lattice structures, torsion, design, additive manufacturing, functionally gradient lattice, torsional stiffness, energy absorption, failure behavior

## Abstract

Functionally graded lattice structures have attracted much attention in engineering due to their excellent mechanical performance resulting from their optimized and application-specific properties. These structures are inspired by nature and are important for a lightweight yet efficient and optimal functionality. They have enhanced mechanical properties over the uniform density counterparts because of their graded design, making them preferable for many applications. Several studies were carried out to investigate the mechanical properties of graded density lattice structures subjected to different types of loadings mainly related to tensile, compression, and fatigue responses. In applications related to biomedical, automotive, and aerospace sectors, dynamic bending and rotational stresses are critical load components. Therefore, the study of torsional properties of functionally gradient lattice structures will contribute to a better implementation of lattice structures in several sectors. In this study, several functionally gradient triply periodic minimal surfaces structures and strut-based lattice structures were designed in cylindrical shapes having 40% relative density. The HP Multi Jet Fusion 4200 3D printer was used to fabricate all specimens for the experimental study. A torsional experiment until the failure of each structure was conducted to investigate properties of the lattice structures such as torsional stiffness, energy absorption, and failure characteristics. The results showed that the stiffness and energy absorption of structures can be improved by an effective material distribution that corresponds to the stress concentration due to torsional load. The TPMS based functionally gradient design showed a 35% increase in torsional stiffness and 15% increase in the ultimate shear strength compared to their uniform counterparts. In addition, results also revealed that an effective material distribution affects the failure mechanism of the lattice structures and delays the plastic deformation, increasing their resistance to torsional loads.

## 1. Introduction

Gradient forms of structures are abundant in nature and are designed to have optimal energy efficiency and adapt well to their ecosystem, making them structurally and functionally optimized [1] for applications including load-bearing and support with high mechanical efficiency, contact damage resistance from loads including impact, indentation and sliding forces, interfacial strengthening and toughening of dissimilar components, and other functional benefits [2]. Some natural examples include the bamboo plant stem [3,4,5], butterfly wings’ scales [6], the nanoporous sucker ring tooth of a squid [7,8], a horse hoof [9], and the spruce stem [10,11]. Generally, the nature of gradients can be in three basic forms. These are gradient in a gradual manner, step-wise manner, or gradient throughout the entire volume [12].

A functional gradient is an essential tool for structural components and lattice structures to control site-specific properties within the structure. It is important in designing structures that mimic natural systems which can have specific and optimal responses to applied loads. A gradient structure can be designed by varying one or more of the following parameters: unit cell size, wall thickness/strut diameter, strut length [1]. The overall procedure taken when designing functionally graded lattice structures includes four main steps [2]: (i) selection or design of the unit cell, (ii) patterning or tessellation of unit cells in the design domain, (iii) relative density distribution for gradient density, and (iv) reconstruction of the lattice structures for manufacturing. Lattice structures can be graded from a complete solid to a minimum beam or wall thickness according to the creation of the desired volume ratio. They can also be manipulated in a variety of ways to control more exotic properties other than density, such as elongation, vibration damping, Poisson ratio, and electromagnetic effects of structures. The manipulation and ability to create very intricate architectures using lattice structures make them extremely difficult to manufacture using conventional fabrication methods. However, Additive manufacturing (AM) processes provide the freedom to manufacture complex parts with the desired accuracy without worrying about the complexity of structures [3]. It provides a wide range of advantages that enable the fabrication of complex functionally gradient structures, including reduced material wastage, the elimination of tooling and fixture preparation, and the reduced cost of labor and machinery. Several AM technologies and processes can also be integrated, forming a high-speed system that is essential to achieve speed, accuracy, and surface finish [4]. This enables engineers to create complex structures that are highly functional without the limitations of manufacturability.

Several studies show that functionally graded lattice structures have superior performance and enhanced properties than uniform density lattice structures. The investigation of the compressive properties of additively manufactured cubic and honeycomb lattice structures showed that plateau stress and the energy absorption of functionally graded structures is higher than the uniform density samples by up to 62% and 72%, respectively [5]. A study performed on continuously graded micro lattices revealed that functional gradient structures exhibit progressive layer-by-layer deformation regardless of unit cell size and build direction favorable for a uni-directional impact absorption compared to uniform density structures that tend to fail diagonally or randomly [6]. The investigation of fatigue properties of additively manufactured gyroid lattice structures showed that graded variants have 1.21–1.67 times higher fatigue life than uniform counterparts having identical volume fractions [7]. Geometrically gradient lattice structures were found to have controllable deformation features and effectively increase the energy absorption efficiency of structures [8]. A quasi-static and dynamic compression test performed in gradient lattice structures manufactured using the SLM process showed that maximum deformation energy depends on the gradient density and increases with increasing relative density [9]. This gradually changing topology resulted in specific mechanical properties, making them good candidates for enhanced energy absorption applications. Studies performed on the impact properties re-entrant auxetic lattice structures applied to a crash box revealed that the gradient lattice structure efficiently improved the crashworthy-ness criteria of the thin-walled columns on pedestrian protection because of their high impact performances [10]. A comparison made between additively manufactured sheet-based and strut-based gyroid cellular structures with graded densities subjected to a compression load showed that the sheet-based structures are more isotropic and have a larger elastic modulus than the strut-based counterparts [11]. It was also proved that the graded cellular structures have excellent deformation and mechanical behaviors, with the sheet-based structures having better energy absorption over the strut-based ones.

Out of the several types of lattice structures, triply periodic minimal surfaces, TPMS, based lattices have improved mechanical properties and are proven to be the most efficient method for creating functionally gradient structures. They have a balanced combination of specific and axisymmetric stiffness [12], high interconnectivity and a high surface-to-volume ratio [13], excellent energy absorption [14], less stress concentration, and increased permeability [15]. Because of their advantages, TPMS structures are strong candidates for different applications in different industries. They have proven to be a crucial and versatile solution for the biomedical sector in designing biomorphic scaffolds because of their lightweight property, as well as porous and interconnected nature, which better mimics the natural human bone structure [16], making them ideal for the fabrication of metal implants [17]. TPMS based metal foam structures have also been used for thermal energy storage and energy management applications because of their effective thermal conductivity [18]. When used in heat exchangers, TPMS structures can generate much larger turbulent kinetic energy, resulting in significantly improved thermal performance [19]. Despite their proven advantages, functionally gradient lattice structures are not fully investigated for dynamic loading conditions such as torsion, shear, and bending. This created a lack of an experimental and analytical model that captures their behavior, limiting the range of their applications.

When creating lattices, unit cells can be tessellated in different ways to make structures [20]. These techniques include the direct patterning of unit cells along two or three dimensions (x, y, z) in the design space, the surface geometry to pattern unit cells conformally, and topology optimization to create lattices to fill a 3D space on the design criteria optimally. All the different techniques of patterning unit cells have their applications and advantages in creating lattice structures. Direct patterning is ideal for quickly and simply creating lattice structures to fill a very regularly shaped design volume. On the other hand, conformal patterning is a vital tool to create lattice structures that conform to a given irregular or curved shape to retain the integrity of unit cells. In contrast, topology optimization is essential because it creates unit cells in a desired 3D space and can optimize material distribution in the lattice structures. Topology optimization is usually suitable for creating stochastic lattice structures based on the stress concentration, boundary constraint, permissible loads, final product weight, and other operating conditions. On the other hand, direct patterning works with non-stochastic unit cells that are capable of being tessellated in two or three dimensions. The conformal patterning method can be used regardless of the lattice being stochastic or non-stochastic. For this study, a circular patterning of unit cells that conforms to the cylindrical design volume is adopted to tesselate unit cells along the direction of the twisting force to study its effect on the torsional performances of structures.

This study aims to design diamond-based and strut-based functionally gradient lattice structures based on the stress distribution and failure of circular structures subjected to torsion in regular and circular patterning methods; investigate their torsional properties in terms of torsional stiffness, energy absorption, and failure modes; and study the effect of circular patterning on the torsional resistance and mechanical properties of the lattice structures. It also gives a performance comparison between functionally gradient structures and their uniform counterparts in terms of their torsional stiffness, ultimate strength, and failure modes.

## 2. Materials and Methods

### 2.1. Design of Structures

**Figure 1:**
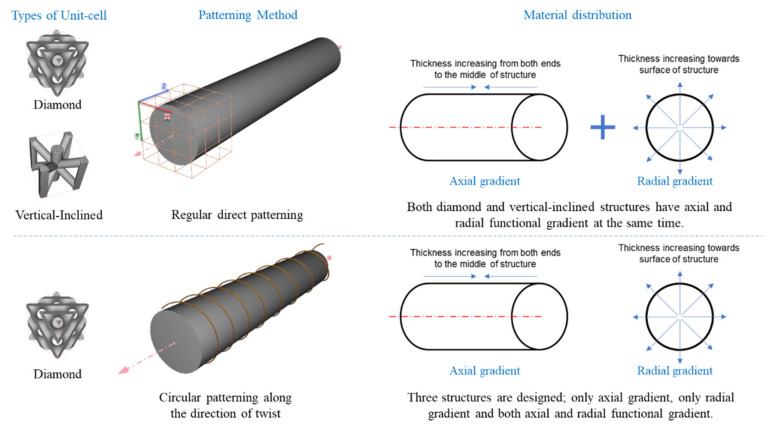
Schematics of functional gradient structures having regular and circular patterning.

When designing complex lattice structures, an implicit modeling approach is necessary to describe these architectures’ geometries precisely. Therefore, nTopology (New York, NY, USA) [21] software is used to design all structures for this study. It uses implicit modeling to represent shapes by using functions rather than b-reps to represent geometries precisely in their true form without initial discretization and by capturing continuity perfectly. Implicit modeling does not rely upon a network of vertices, edges, or faces, making it significantly lighter to compute and design complex structures. Other advantages of nTopology include the ability to use a field-driven approach to increase flexibility and efficiency. Using different field functions, it is possible to manipulate the topology of structures and make functionally gradient designs as per design requirements. In this study, five different structures are designed in a cylindrical design domain according to the standard ASTM E143-13 Standard Test Method for Shear Modulus at Room Temperature [22]. Two of the designs are diamond and vertical-inclined unit cells with a regular direct patterning of unit cells. The remaining three designs follow a circular patterning of unit cells along the length of the structure, as shown in Figure 1 below.

**Figure 2:**
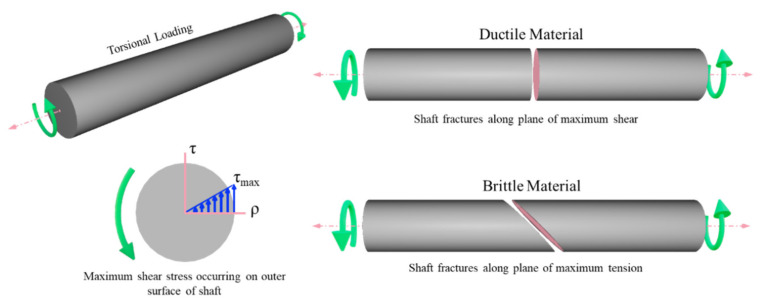
Stress concentration in torsional loading and failure of ductile and brittle materials.

For a cylindrical-shaped structure subjected to torsional loading, the shear stress varies linearly with the radial distance with the maximum shear stress occurring at the surface of the shaft. Therefore, for cylindrical structures subjected to torsion, there will be a high stress concentration on members on the outer surface of the structure. For ductile materials, failure due to torsion occurs along the plane of maximum shear stress, usually perpendicular to the structure’s axis. On the other hand, brittle materials tend to fail along the plane of maximum tensile stress, which is at an angle to the structure’s axis. In both cases, the maximum stress concentration is at the structure’s surface and closer to the middle of the structure. Figure 2 illustrates the stress concentration and failure due to torsion in circular elements subjected to torsion.

**Figure 3:**
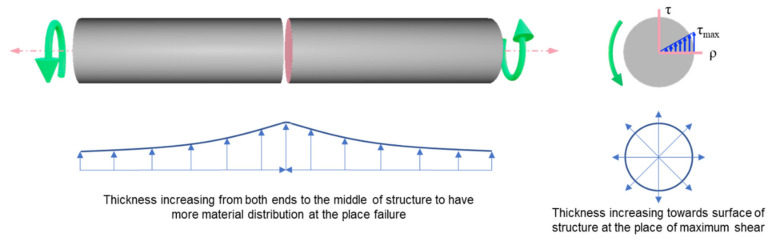
Schematic representation of functionally gradient lattice structures.

As illustrated in the above figure, maximum shear stress occurs at the surface of the structures, and failure occurs at the middle of the shaft for both ductile and brittle materials. Therefore, functionally gradient lattice structures have a material distribution driven by high-stress concentration areas for cylindrical structures subjected to torsion. Figure 3 shows the schematic representation of functional gradient structures designed for this study.

**Figure 4:**
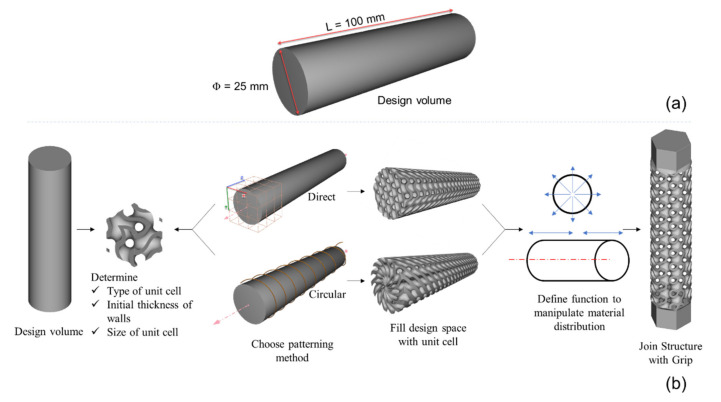
(**a**) Design volume used for all structures, (**b**) Overall procedure used to design functionally gradient lattice structures.

The overall dimensions of the structures are shown in Figure 4 below. The length of the structures is 100 mm without including grips, and the diameter is 25 mm. The hexagonal grips at both ends of the structures have a length of 16 mm inscribed in a 25 mm circle. These grips are designed to fit the hexagonal socket fixture used to hold the specimen in the grips of the torsion testing machine. Figure 4 shows the overall dimensions and design procedures used to design the lattice structures for this study. The two structures with regular direct patterning having a functional gradient in both axial and radial direction made from diamond and vertical-inclined unit cells are referred to as D and VI, respectively. The structure with circular patterning with only an axial functional gradient is called CA; the structure with circular patterning with only a radial functional gradient is called CR. The structure with circular patterning having both axial and functional gradients is referred to as CF.

**Figure 5:**
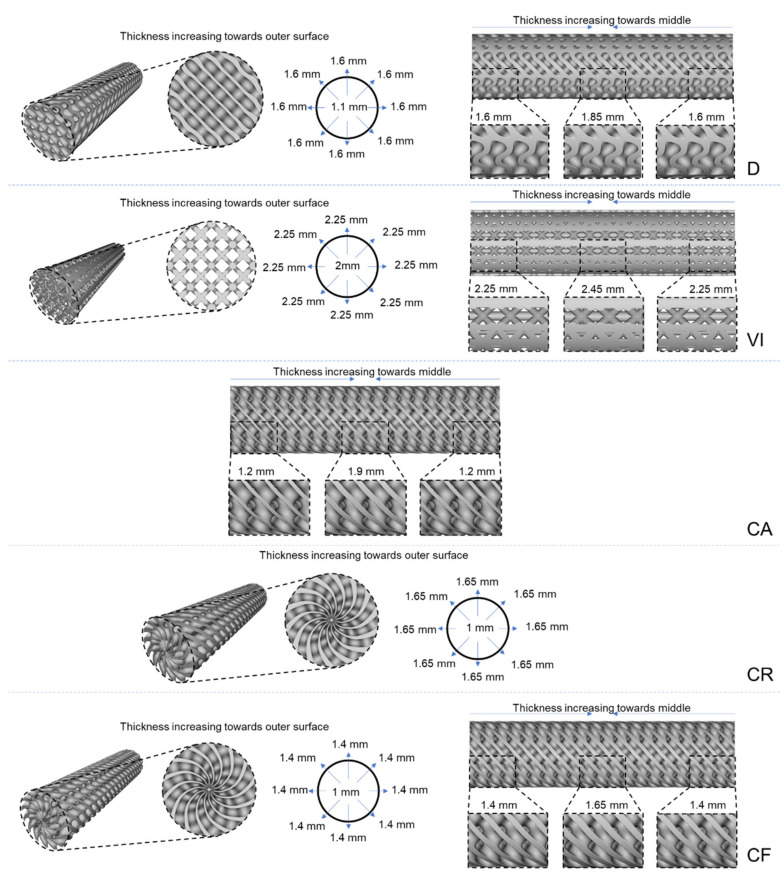
Longitudinal and radial density gradient in five functional gradient designs.

The general overall procedure to design all structures includes using TPMS blocks with an offsetting ramp function to drive their material distribution in the desired radial or longitudinal direction with the required wall thickness. All structures were designed with around 40% relative density. Structure D is designed with an initial unit cell thickness of 1.1 mm and 10 mm × 10 mm × 10 mm unit cell size. A radial thickness variation that varies from 1.1 mm from the central axis to 1.6 mm to the outer surface is achieved using an offset body function. This radial variation is later used to create a longitudinal density gradient which increases from 1.6 mm from the left and right end to 1.85 mm towards the middle of the structure. The second structure, VI, starts having four outer vertical struts having 2 mm, a middle vertical strut of 1.6 mm, and inclined struts of 1.2 mm diameter. Using the ramp function, a radial thickness variation that increases from 2 mm to 2.25 mm towards the outer surface is created, which is then used to create a longitudinal variation in which the unit cell thickness increases to 2.45 mm toward the middle of the structure from the left and right ends. The CA structure only has a longitudinal density gradient which increases from the left and right ends towards the middle from 1.2 mm to 1.9 mm. On the other hand, the CR structure only has a radial density gradient of 1 mm at the central axis and increases linearly to 1.65 mm towards the outer surface. The final structure, CF, combines both longitudinal and radial density gradients. The radial variation has a unit cell thickness that increases from 1 mm to 1.4 mm from the central axis to the outer surface, and the longitudinal density gradient increases from 1.4 mm, from the left and right ends, to 1.65 mm to the middle of the structure. Figure 5 shows all the density gradients and unit cell thicknesses discussed in the above paragraph.

**Figure 6:**
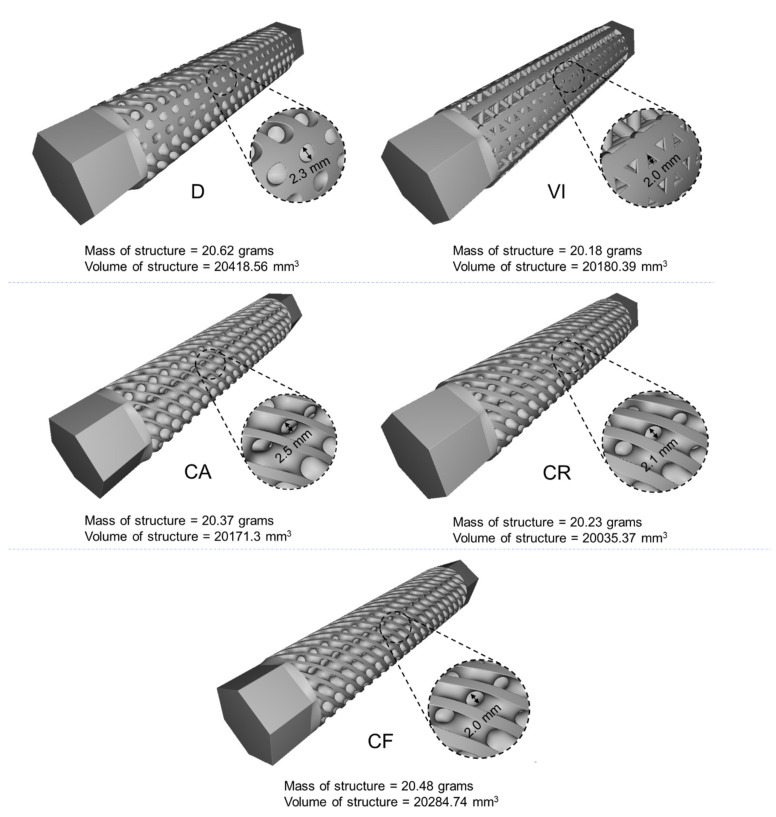
Mass, volume, and minimum pore size of the five functional gradient structures.

After designing each structure, inspections blocks are used to calculate the mass, volume, and minimum pore size of structures to ensure all structures have similar masses and an outside pore size that will allow powder removal without difficulty. Figure 6 shows all five designs joined with hexagonal grips and their mass volume and minimum pore size. It shows that all structures have very close values to compare their performances.

Table 1 below shows the unit cell size, wall thickness, relative density, and minimum pore size of the structures designed for this study. From this table, we can see that all structures are designed with the same relative density.

### 2.2. Additive Manufacturing of Structures

**Figure 7:**
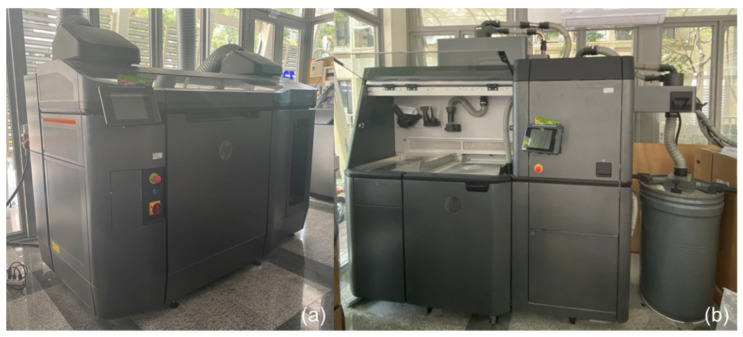
(**a**) HP MJF 4200 printer, and (**b**) HP MJF Post-processing station.

All samples of lattice structures are manufactured using a high-speed HP Multi Jet Fusion (MJF) 4200 series 3D Printer [23]. This additive manufacturing machine uses a technology that is a hybrid between powder bed fusion (PBF) and binder jetting (BJ) processes. These two technologies are among the most common processes used for polymer and metal 3D printing technology. The main difference between these two technologies is how the geometrical shape of the printed part is achieved. The HP MJF technology creates layers by depositing a fusing agent on a powder bed, such as BJ, and uses a heating source to fuse powder particles, such as PBF, and form parts that are final and do not require further sintering in a furnace. Each specimen is fabricated using a Nylon Polyamide 12 (PA 12) powder, a robust thermoplastic material that produces high-density parts with balanced profiles and robust structures. PA 12 material has excellent mechanical and elastic properties [24] and provides good chemical resistance to oils, greases, aliphatic hydrocarbons, and alkalis. It is also ideal for complex assemblies, housings, enclosures, and watertight applications. This material, combined with HP MJF solutions, can achieve a low cost per part and minimized waste due to its balanced reusability and performance characteristics. Figure 7 shows the HP MJF 4200 printer and post-processing station used for this study.

**Figure 8:**
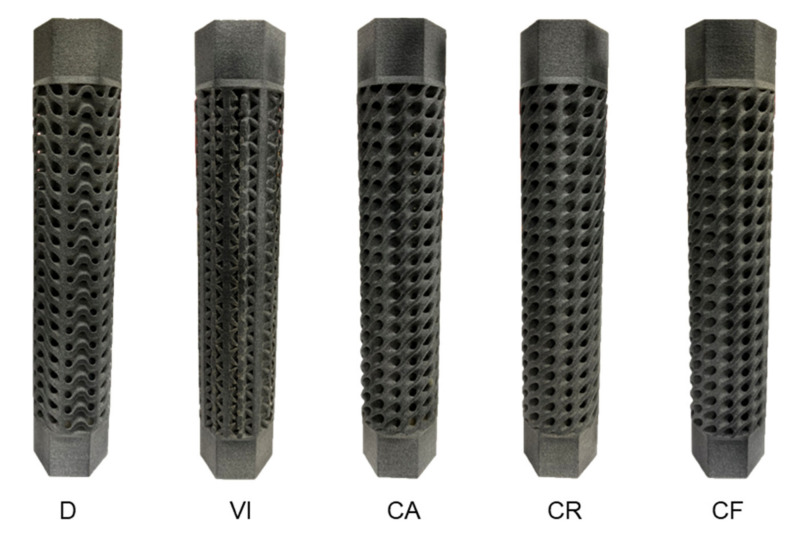
Additively manufactured samples of each lattice structure.

For the torsional experiment, three samples for each design are manufactured. All the printed samples are individually weighed and also measured for their dimensional accuracy. The tolerances of grip structures for holding fixtures are also tested and proven to be accurate enough. All dimensions, as well as weight, are compared with initial design values. Almost all samples printed for each lattice structure have printed weights slightly less than design weights. This is due to the inherent porosity of 3D printing technologies and the inability to achieve 100% dense parts. On the other hand, a few specimens have printed weights slightly larger than design weights due to unremoved powder particles in the sandblasting process due to the complexity of structures. Figure 8 shows one specimen from each of the five functionally gradient lattice structures. It can be seen from the figure that printed parts have good quality.

The summary of weights of printed specimens and a comparison with CAD values is shown in Table 2 below.

### 2.3. Experimental Setup

**Figure 9:**
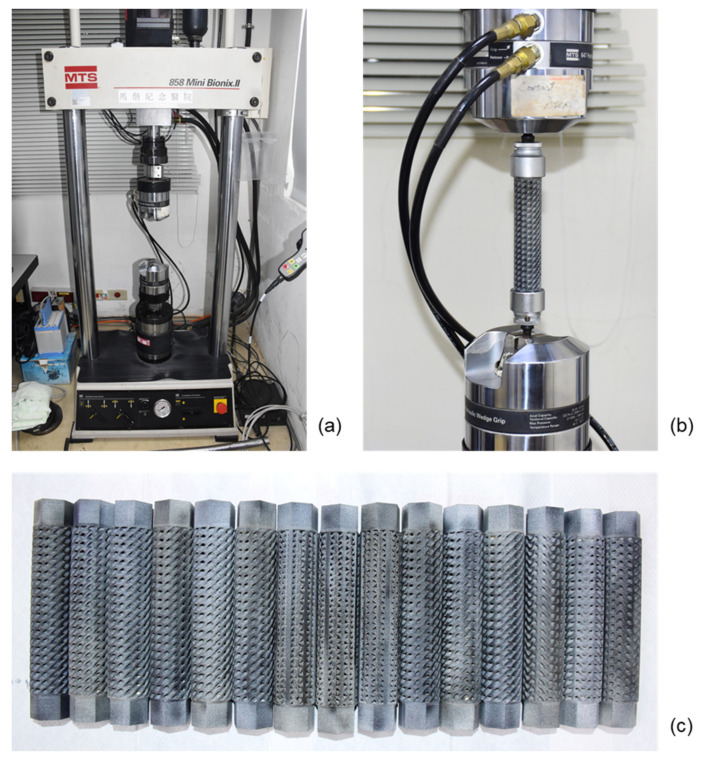
(**a**) Torsion testing machine, (**b**) Experimental setup and fitting of specimen in the machine, and (**c**) All three specimens for each lattice structure.

The torsion experiment is carried out using the Material Testing System machine (Mini MTS 858, MTS Corporation, Minneapolis, MN, USA). The test is carried out at a speed of 30 degrees/min, which is high enough to make creep negligible [25]. Figure 9 below shows the test equipment, experimental setup for torsional testing, and specimens of the structures.

From the experiment, raw values of time (s), torsional angle (rad), and torque (N·mm) are continuously and automatically recorded on a computer that is directly connected with the test machine using the software Multipurpose Elite Software (MTS Corporation, Minneapolis, MN, USA). These values are later used to calculate several mechanical properties and torsional properties of structures.

## 3. Results and Discussion

The torsional properties of all lattice structures in terms of torsional stiffness, energy absorption, and failure modes are presented in the coming sections.

### 3.1. Torsional Stiffness

**Figure 10:**
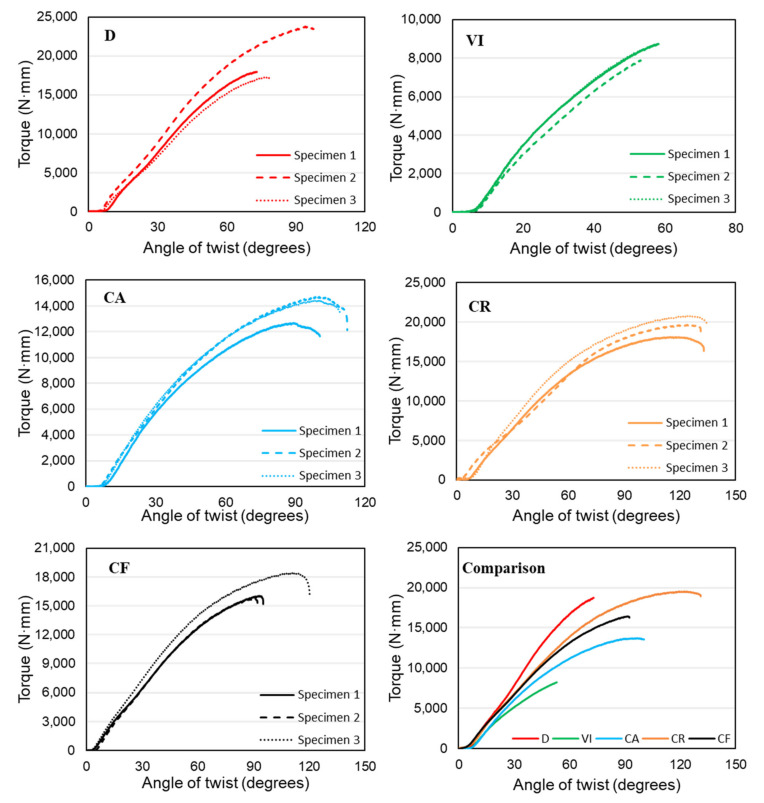
Experimental Torque-Twist curves of functionally gradient lattice structures.

The torque-twist plots for the five functionally gradient structures are shown in Figure 10 below.

The first design, D, has a functional material distribution similar to a stress concentration along the radius of a cylindrical object subjected to torsion and where structures fail, which is the middle of the structure. Because of this, we see this structure having steeper curves and higher torque values. This structure has regularly patterned unit cells in the design volume. Another structure with the regularly patterned unit cells is design VI. This has a similar functional gradient to the first structure, both along the radial and longitudinal directions. This structure is composed of vertical struts that are far less efficient in resisting torsional loads. Because of this reason, this structure withstood significantly less torque.

The other three structures have a circular patterning of unit cells which corresponds to the direction of the twisting load. This made the structures resist more angular twists than the other two functional gradient structures. It can also be seen from the figure below that these three lattice structures have larger plastic deformation regions characterized by a significant increase in the angular twist with a meager increase in the amount of torque. However, we can see that the CF structure that has a functional gradient in both the radial and longitudinal direction was able to resist lower torque value than the regular patterned D structure with the same radial and longitudinal functional gradient design, showing that a regular patterning of unit cells is better than a circular patterning for torsional applications. Structure D has an average torque value of 19,693 N·mm, which CR follows with 19,536 N·mm. These two structures have similar average torque values, but CR withstood much more angular twisting of around 130 degrees compared to just 80 degrees by structure D. These structures are followed by CF, CA, and VI with 16,739, 13,942, and 8411 N·mm, respectively.

**Figure 11:**
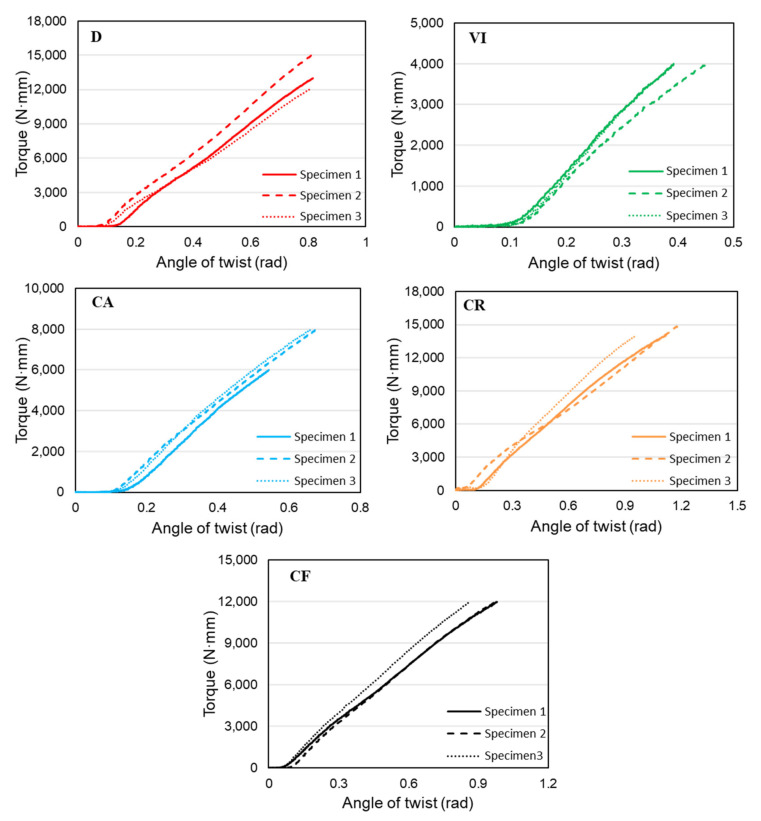
Elastic regions of structures for calculation of torsional stiffness.

The elastic regions of these torque-twist plots are shown in Figure 11 below and are used to calculate the torsional stiffness of structures. Even though the functionally gradient structures did not exhibit large angular twists as in the previous designs, they showed higher stiffness values because of their effective material distribution. Based on the elastic curves of torque-twist plots, torsional stiffness values for each specimen are calculated, and the average value is taken as the torsional stiffness of the structures. The individual and average values of torsional stiffness for each structure are presented in Table 3. The table shows that design D has the highest stiffness value of 18,705 N·mm/rad due to its effective material distribution across the entire structure. It is followed by a CR structure with 14,220 N·mm/rad, which has a material distribution that follows a natural stress concentration in torsional load. Even though the CF structure has a functional gradient design in both the radial and longitudinal directions, it has a slightly lower stiffness value, showing that the radial stress concentration is a higher determining factor in the torsional performances of cylindrical structures. The CA and VI structures follow with stiffness values of 13,812 and 12,703 N·mm/rad, respectively. The difference in the torsional stiffness of the structures can also be seen from the slope of the curve in the previous figure showing the comparison between the torque-angle of the twist curves.

### 3.2. Energy Absorption

To calculate the energy absorptions of structures polar from the shear stress-strain curves, the polar moment of inertia of the structures is calculated by taking multiple slices of the cross-section to determine the cross-section with the smallest area that can better represent the torsional resistance of the entire structure. This cross-section is then used to calculate the polar moment of inertia at the centroid of the area. Table 4 shows the polar moment of the inertia value for each structure based on their minimum cross-sectional area.

**Figure 12:**
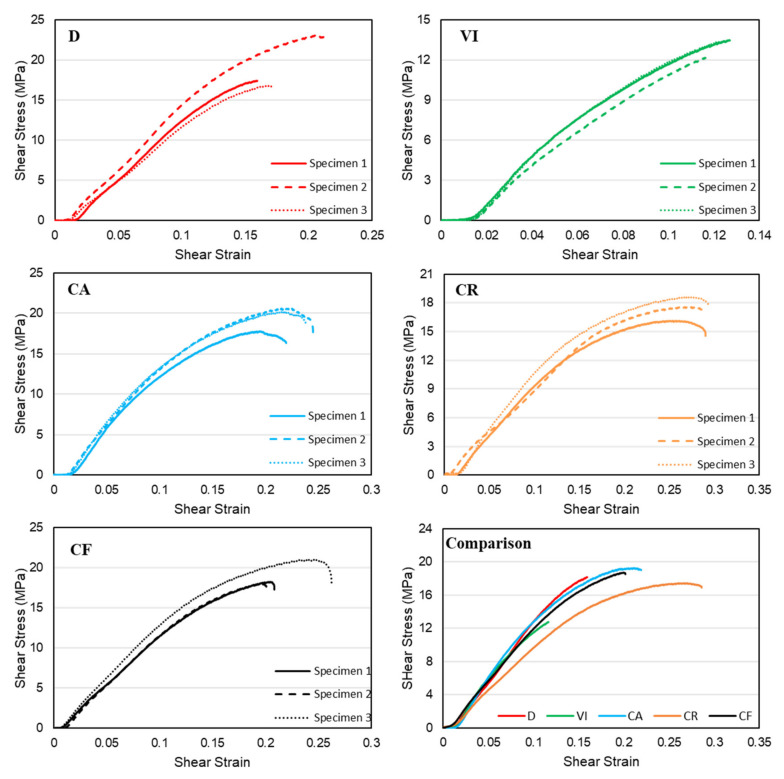
Experimental shear-stress-strain curves of functionally gradient lattice structures.

Since the polar moment of inertia is directly proportional to the cross-sectional area, we can see on the above table that structures with higher areas will have higher values of the polar moment of inertia. Once the polar moment of inertia is determined, the shear stress and strain values of each structure can be calculated from the torque-twist value using Equations (1) and (2), respectively.

(1) $$\mathrm{Shear}\;\mathrm{stress}—\tau =\frac{T\times r}{J}$$

(2) $$\mathrm{Shear}\;\mathrm{strain}—\gamma =\frac{r\times \theta }{L}$$

where T = torque (N·mm), r = radius (mm), J = polar moment of inertia (mm^4^), θ= angle of twist (rad), and L = gauge length (mm). Since the radius value is constant for all the structures, the value of shear stress mainly depends on the amount of torque and the value of the polar moment of inertia of the structures. Figure 12 shows the experimental shear-stress-strain plots of the five functionally gradient structures. Three structures, D, CA, and CF have very close maximum shear stress values of 19.11, 19.88, and 19.06 MPa. These structures have higher stress values than the two other structures. Since structure CR had a slightly higher polar moment of inertia value, it decreased the maximum shear stress it withstood with a value of 17.43 MPa and put it just less than the three previously mentioned designs. Structure VI had the lowest value of maximum shear stress with 13.01 MPa and a lower shear strain value. We can also see from the figure that the two structures with a regular patterning of unit cells, D and VI, had very short regions of plastic deformation compared to the three circularly patterned lattice structures.

**Figure 13:**
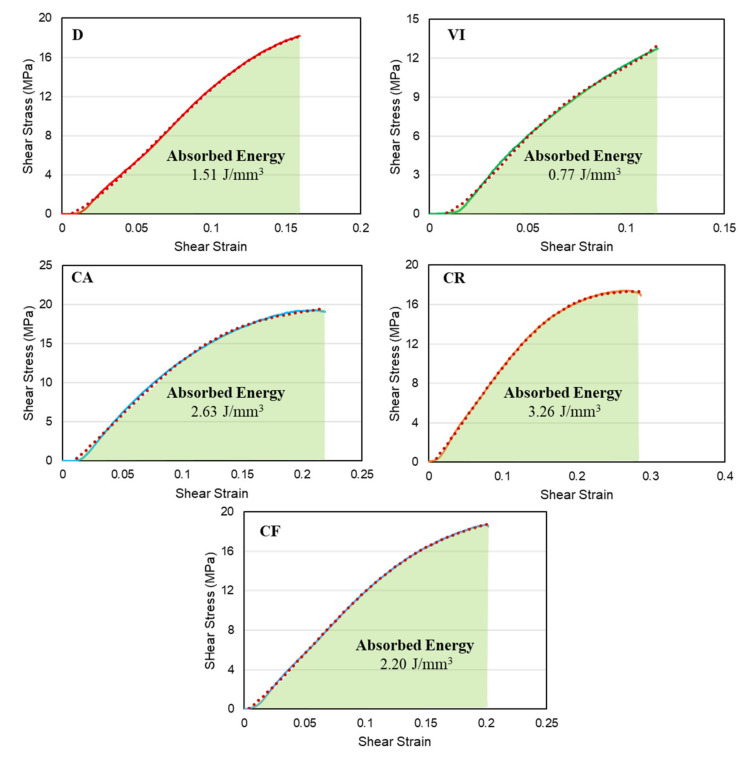
Energy absorption value based on the average stress-strain result of lattice structures.

The energy absorption of each structure is calculated by taking the area under the average shear-stress-strain curves. The energy absorbed up to the fracture point of the specimens, toughness, is calculated by approximating the stress-strain curves using the best polynomial curve and integrating the function with the range of the lowest and highest value of strain. Figure 13 shows energy absorption plots based on the area under the shear-stress-strain curves of each lattice structure. The figure below shows that the CR structure has the highest energy absorption value of 3.26 J/mm^3^ due to its high angular twist values. It is followed by the CA, CF, D, and VI structures with energy absorption values of 2.63, 2.20, 1.51, and 0.77 J/mm^3^, respectively. The circular patterning of the three structures, CR, CA, and CF, gave them the advantage to withstand more angular twist over the regularly patterned unit cells.

### 3.3. Comparison of Performance

**Figure 14:**
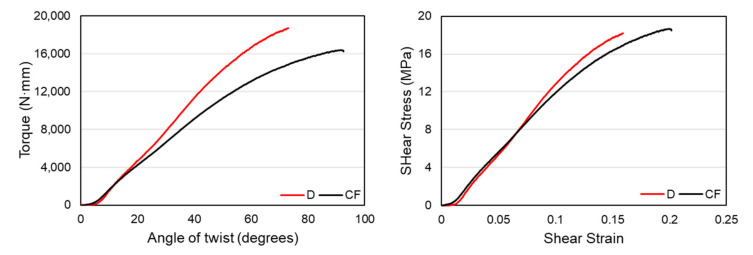
Comparison of torque-twist and shear stress-strain curves between D and CF structures.

In this section, the comparison between regularly patterned and circularly patterned diamond structures will be made in terms of their torsional stiffness, ultimate strength, and angular twist. In addition, a comparison of the performance between the regularly patterned functional gradient diamond and vertical-inclined structures is presented to show the effect of the material distribution on the torsional performance of structures. For a comparison of the torsional stiffness of structures, the elastic regions of the torque-twist curves were plotted using the average values of the three specimens. The first structures that are compared in this section are structures D and CF. Both structures have a material distribution that varies in both longitudinal and radial directions in which the wall thickness of the structures increases from the central axis to the outer surface and from two longitudinal ends towards the middle. Figure 14 below shows the torque-twist and shear stress-strain curves of the D and CF structure. The figure shows that the regularly patterned functional gradient structure, D, has a higher torsional resistance than the circularly patterned CF structure.

The curve also has a much steeper curve in the elastic region, showing a better torsional stiffness value. In contrast, the CF structure has a higher value of angular twist due to the patterning effect that aligns to the twist’s direction, giving it better compliance to the twisting load. From the stress-strain plot, we can see that both D and CF structures have comparably similar ultimate strength values of 18.16 and 18.68 MPa, respectively. However, the CF structure was able to withstand higher shear strain values deforming plastically. The torsional stiffness values of both structures are shown in Table 5 below.

**Figure 15:**
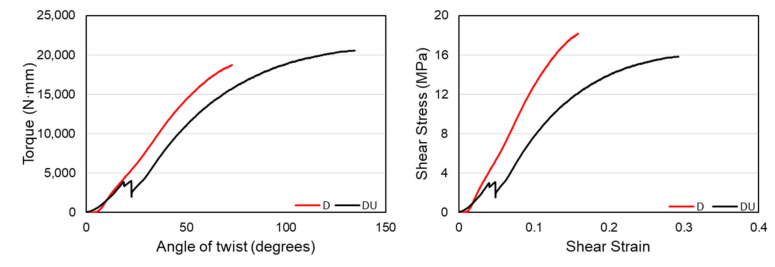
Comparison of torque-twist and shear-stress-strain curves between D and DU structures.

Another comparison is made between uniform density structures and functionally gradient structures. The uniform gradient regularly patterned diamond structure, hereafter DU, is made with a wall thickness of 1.72 mm and a relative density of 40.18%, similar to the functional gradient counterpart. Similar torsional experiments with identical parameters and conditions are made to compare the properties between the two structures. On the other hand, the uniform density vertical-inclined structure, hereafter VI-U, has an outer vertical beam diameter of 2.4 mm and diagonal beams of 1.6 mm. This structure also has a similar relative density of 41% with the functional gradient one. Figure 15 shows a comparison of torque-twist and shear stress-strain plots between the gradient density D, structure, and the uniform density, DU, counterpart.

The above graph shows that the uniform density DU structure has higher values than the functionally gradient D structure in terms of the torque withstood and torsional angle. One major factor for these results is that both structures have the same relative density, making the D structure have less wall thickness at the end of the structures, creating a high-stress concentration in those areas. However, structure DU does not have any variation of material distribution in the longitudinal direction that eliminated a high-stress concentration at the intersection of the structure and grip. The shear-stress-strain plots show that the functionally gradient D structure has a very steep slope and higher ultimate strength than the uniform gradient DU structure. This resulted from the D structure’s torsional resistance values governed by the material distribution along with the structures. Since the material distribution follows a stress concentration pattern in cylindrical structures, it was able to have higher torsional stiffness. The torsional stiffness values of these two structures are given in Table 6 below.

**Figure 16:**
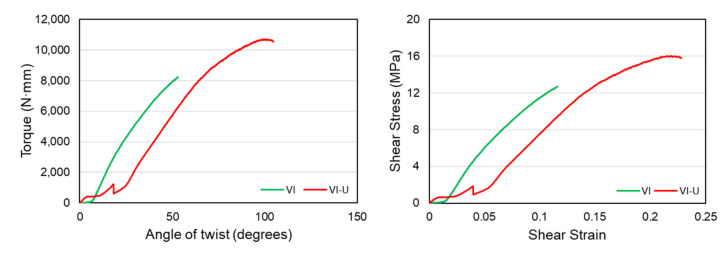
Comparison of torque-twist and shear-stress-strain curves between VI and VI-U structures.

Similarly, a comparison is made between the functionally gradient and uniform density vertical-inclined structures. The comparison of the torque-twist and shear-stress-strain plots is given in Figure 16 below. The figure shows that the uniform density vertical-inclined VI-U structure could withstand more angular twists than the VI structure. This is mainly caused due to the higher stiffness of the structure, which made it behave in a brittle manner and have failure without having significant plastic deformation. Therefore, we can see that the different material distribution in the structures made from the same unit cell can significantly affect material failure behavior. In terms of the shear-stress-strain plots, structure VI-U still has higher ultimate shear strength before failure. However, the functionally gradient structure, VI, has a smooth and slightly steeper curve showing a higher stiffness value. Overall, we can say that material distribution is more efficient in the diamond structure to enhance the property than the strut-based vertical-inclined structure.

The torsional stiffness values of these two structures, VI and VI-U, are given in Table 7 below. The table shows that the torsional stiffness of the VI structure is slightly higher than the VI-U structure despite not having a large plastic deformation region.

### 3.4. Failure of Structures

**Figure 17:**
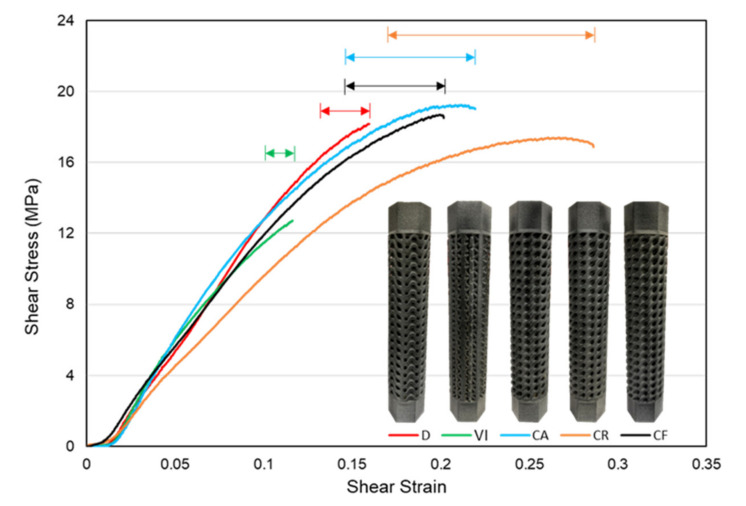
Approximate plastic deformation regions based on stress-strain curves of structures.

Figure 17 shows the plastic regions of each structure based on the shear-stress-strain curves. It shows that the three structures with a circular patterning of unit cells, CA, CR, and CF, in the design volume showed larger plastic deformation regions. This is because the circular patterning matches the twisting profile of the angular twist, giving them a higher capacity to withstand more torsional angles. In contrast, the two functionally gradient structures with a regular patterning of unit cells, D and VI, have shorter plastic deformation regions. The amount of plastic deformation before fracture can predict the type of failure behavior in structures. Structures with more extended regions of plastic deformation are often associated with the ductile mode of failure. This shows that CA, CR, and CF structures failed in a brittle manner. Since D and VI structures have stiff structures and shorter plastic deformation regions, we can say that they failed in a brittle manner.

The failed specimens can also indicate the mechanical properties of the structure and their resistance to the applied torsion.

**Figure 18:**
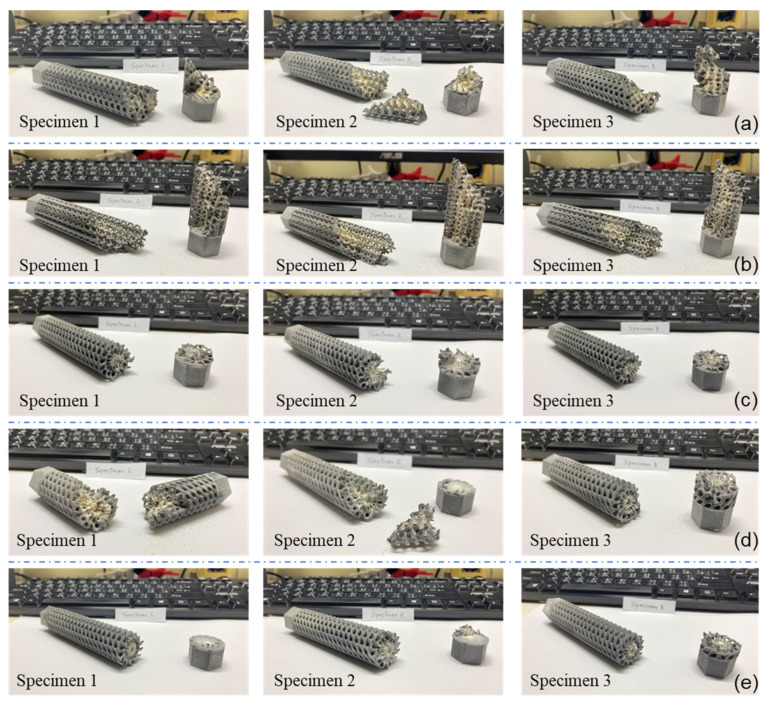
Mode of failure for different functionally gradient design configurations investigated in the present study. (**a**) D, (**b**) VI, (**c**) CA, (**d**) CR, and (**e**) CF.

Figure 18 below shows all failed specimens of the functionally gradient lattice structures. The stiff structures were able to withstand the applied torsional rotation failed at the interface between the lattice and the grip structure. This shows that the interface was subjected to higher stress values due to the higher strength of the main structure in the functionally gradient structures. On the other hand, it can be seen that the failure was initiated from the middle of the structures where the stress concentration was higher for the uniform density structures. This shows that the functional material distribution effectively made the lattice structures stiffer and have better torsional resistance.

## 4. Conclusions

This paper aimed to investigate the torsional properties of lattice structures and study the effect of the material distribution and patterning modes on the mechanical properties of the structures. The torsional properties of functionally gradient lattice structures made from TPMS and strut-based unit cells were investigated using an experimental approach. The results clearly show that the stiffness and energy absorption of the structures can be improved by an effective material distribution that corresponds to the stress concentration due to torsional load. It is observed that functional gradient designs have higher stiffness, increasing their strength. However, this increase in stiffness is seen to be associated with a more brittle mode of fracture. In addition, effective material distribution also affects the failure mechanism and delays the plastic deformation of the structures, increasing their resistance to torsional loads. The functionally gradient lattice structures’ results showed that structure D with a functional material distribution in both radial and longitudinal directions had improved torsional stiffness and ultimate shear strength compared to structure DU, a uniform density structure. On the other hand, the higher stiffness of this structure made it have shorter plastic deformation failing in a brittle manner. It is also found that the functionally gradient structure made from vertical-inclined unit cells, VI, has higher torsional stiffness than its uniform density counterpart, VI-U. A circular way of patterning unit cells was also used to investigate its effect on torsional properties. From the results, it can be concluded that this was of patterning unit cells increased the amount of the torsional twisting angle that a structure can withstand but has no importance in enhancing torsional stiffness and the ultimate shear strength of the structures.

For future work, it is recommended that an extensive finite element analysis, FEA, be carried out, and cross-validation with experimental results should be conducted to characterize the lattice structures’ torsional properties effectively. This study can open further avenues to explore functionally graded structures by the optimal distribution of materials in order to achieve the specific strength needed for application where weight is a significant factor, and further investigation that looks at the dynamic underlying reasons in the mechanics of these structures with accurate methods of evaluating them will create a potential to exploit them further and expand the impact of TPMS based lattice structures.

## Figures and Tables

**Table 1:** Summary of dimensions for the five functionally gradient structures.

Design	Unit Cell Size (mm)	Wall Thickness (mm)	Relative Density (%)	Minimum Pore Size (mm)
D	10 × 10 × 10	1.6–1.85	41.59	2.3
VI	6 × 6 × 6	2.25–2.45	41.11	2.0
CA	7, 10 × 10	1.2–1.9	41.09	2.5
CR	7, 10 × 10	1.0–1.65	40.81	2.1
CF	7, 10 × 10	1.25–1.65	41.32	2.0

**Table 2:** Weight of printed samples and comparison with CAD value.

Design	Weight (g)				Specimen Average (g)	The Difference with CAD Value
	CAD	Specimen 1	Specimen 2	Specimen 3		
D	33.7	31.7	31.8	31.2	31.6	6.4%
VI	33.2	31.2	31.1	31.3	31.2	6.2%
CA	33.5	31.4	33.1	32.7	32.4	3.3%
CR	33.4	32.0	33.0	33.4	32.8	1.8%
CF	33.6	32.42	32.56	32.67	32.5	3.3%

**Table 3:** Individual and average torsional stiffness values of structures.

Design	Individual Torsional Stiffness (N·mm/rad)			Average Torsional Stiffness, T/θ (N·mm/rad)
	Specimen 1	Specimen 2	Specimen 3	
D	18,382	20,875.11	16,856.9	18,704.67
VI	13,367.66	11,287.73	13,454.63	12,703.34
CA	13,528.55	13,762.95	14,145.4	13,812.3
CR	13,664.31	12,941.55	16,054.45	14,220.11
CF	13,073.77	13,655.06	14,852.21	13,860.35

**Table 4:** Polar moment of inertia values based on the minimum cross-sectional area of structures.

Design	Minimum Cross-Sectional Area (mm^2^)	Polar Moment of Inertia (mm^4^)
D	144.39	12,883.02
VI	96.76	8083.33
CA	125.45	8915.24
CR	162.7	14,007.22
CF	135.53	10,977.03

**Table 5:** Torsional stiffness values of D and CF structures.

Design	Torsional Stiffness, T/θ (N·mm/rad)
D	18,704.7
CF	13,860.35

**Table 6:** Torsional stiffness values of D and DU structures.

Design	Torsional Stiffness, T/θ (N·mm/rad)
D	18,704.7
DU	18,162

**Table 7:** Torsional stiffness values of VI and VI-U structures.

Design	Torsional Stiffness, T/θ (N·mm/rad)
VI	12,703.34
VI-U	9990.2

## Data Availability

Not applicable.
